# Association of neutrophil-to-lymphocyte ratio with severe abdominal aortic calcification: new evidence from the United States

**DOI:** 10.3389/fcvm.2025.1496552

**Published:** 2025-02-11

**Authors:** Hao Deng, Mengmeng Sun, Zhentong Zhao, Kun Fan, Zizhang Zhao, Yifan Chi, Wei Sheng

**Affiliations:** ^1^ Department of Heart Center, QingDao Hiser Hospital Affiliated of Qingdao University, QingDao, ShangDong, China; ^2^ Department of Thyroid Surgery, QingDao Municipal Hospital, QingDao, ShangDong, China

**Keywords:** abdominal aortic calcification, atherosclerosis, inflammation, neutrophil-tolymphocyte ratio, retrospective study

## Abstract

**Background:**

Abdominal aortic calcification (AAC), an early indicator of abdominal aortic wall atherosclerosis, is a marker of subclinical atherosclerosis and a predictive factor for vascular-associated morbidity and mortality. These outcomes are driven by inflammatory processes. Given the pivotal role of inflammatory mediators in the pathogenesis of aortic calcification, inflammation has attracted interest as a peripheral biomarker for early mortality prediction in patients with aortic calcification. The neutrophil-to-lymphocyte ratio (NLR) measured in the peripheral blood typically reflects the body's inflammatory response by combining laboratory markers of innate and adaptive immunity. The NLR is associated with the prognosis of a range of diseases, including circulatory, infectious, psychiatric, and neoplastic conditions. However, the precise relationship between the NLR and vascular calcification remains unclear. Therefore, the present study investigated the correlation between the NLR and AAC in a nationally representative sample from the US.

**Methods:**

This study analyzed data from the National Health and Nutrition Examination Survey (NHANES) 2013–2014. Multivariable logistic regression, stratified analysis with interaction, and restricted cubic spline analysis were used to examine the relationship between the NLR and AAC.

**Results:**

This study enrolled 3,047 participants [1,469 men (48.2%) and 1,578 women (51.8%)]. After adjusting for all covariates in the multivariate logistic regression, an independent association was identified between augmented NLR and the incidence of severe AAC (SAAC). The risk of SAAC increased by 8% with every 1% increase in NLR. Compared with the lowest NLR group [quartile 1 (Q1), <1.64], the adjusted odds ratio values for NLR and SAAC in Q3 (>2.34) were 1.42 (95% confidence interval: 1.02–1.96, *p* = 0.037), respectively. The results of subgroup analyses revealed no significant interaction effects.

**Conclusions:**

The NLR was positively correlated with SAAC prevalence among adults in the US. These findings have significant clinical relevance and may inform clinicians regarding the management of SAAC. However, further research is required to confirm this association.

## 1 Introduction

Vascular calcification (VC) is a pathological process involving hydroxyapatite mineral deposition in the vascular system (1). VC correlates strongly with a range of cardiovascular diseases, including those affecting the thoracic and abdominal aorta. Abdominal aortic calcification (AAC) is an early indicator of aortic wall atherosclerosis and is typically driven by inflammatory processes (2). Thus, AAC may be a marker of subclinical atherosclerosis and a predictor of vascular-related morbidity and mortality (3). Nevertheless, the complete pathogenesis of VC remains unclear, and no specific preferred treatment approach has yet been established (4). Therefore, a deeper understanding of the factors that influence VC and implement effective preventive measures is urgently needed. Given the pivotal role of inflammatory mediators in the pathogenesis of aortic calcification, increasing interest has been paid to utilizing this process as a peripheral biomarker for early mortality prediction in patients with aortic calcification (5).

Neutrophils and lymphocytes are primary inflammatory cells in peripheral blood. Their ratio, that is, the neutrophil-to-lymphocyte ratio (NLR), is a reliable biomarker of systemic inflammatory status. Accumulating evidence (6) has focused on the important effects of NLR on individuals at high cardiovascular risk. A study found (7) that the higher the NLR, the higher the cardiovascular risk, which in turn is closely related to the progression of target organ damage and arteriosclerosis (8). Neutrophils can aggravate endothelial cell injury by releasing inflammatory mediators and oxidative stress factors, leading to the deterioration of vascular function and the formation of atherosclerotic plaques (9). Lymphocytes are important in the formation of inflammation and immune regulation. Therefore, elevated NLR may indicate an increased inflammatory state, increased vascular damage, and an increased risk of cardiovascular death. In this context, the NLR may be a novel inflammatory biomarker for the prognosis of numerous cardiovascular diseases.

Although AAC is an inflammatory process, the precise relationship between the NLR and AAC remains unclear, meriting further investigation. To address this research gap, our primary objective was to deepen our understanding of how NLR influences AAC and the implications of this association. Established evidence among adults in the U.S. is scarce. Thus, our study aimed to provide additional insights into the interplay between the NLR and AAC. Therefore, this retrospective cross-sectional study analyzed data from 3,047 participants from the National Health and Nutrition Examination Survey (NHANES) 2013–2014 cohort.

## 2 Materials and methods

### 2.1 Study population

The NHANES is a nationally representative survey conducted by the National Center for Health Statistics (NCHS) that employs stratified, multistage probability cluster sampling to assess the health or nutritional status of the non-institutionalized US population (10). The sample for the survey was selected as a representative of the U.S. population across ages. Information on AAC was provided only in the NHANES 2013–2014 cycles for adults aged 40–80 years.

**Figure 1:**
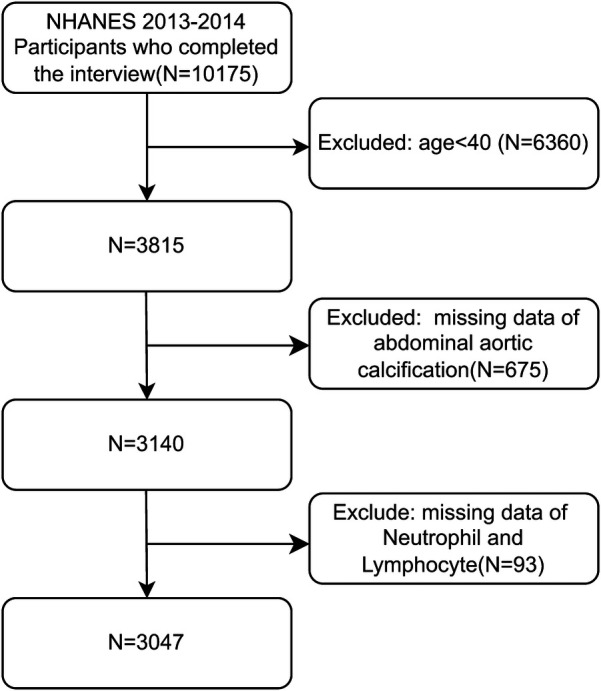
Flowchart of this study.

From the initial pool of 10,175 individuals in the 2013–2014 NHANES cycle, we excluded those <40 years of age (*n* = 6,360), with missing AAC scores (*n* = 675), and with incomplete neutrophil and lymphocyte data (*n* = 93), resulting in a final sample of 3,047 participants (Figure 1).

The NHANES protocol was approved by the NCHS Research Ethics Review Board (11), and written informed consent was obtained from all participants at the time of enrollment. The Ethics Committee of Hiser Hospital, affiliated with Qingdao University, determined that the present study was exempt from review because it used publicly available de-identified data and waived the requirement for informed consent. This study was conducted in accordance with the Strengthening the Reporting of Observational Studies in Epidemiology (STROBE) Reporting Guidelines.

### 2.2 Study variables

#### 2.2.1 NLR

The NHANES and complete blood count (CBC) profiles were used to acquire hematologic parameters. The CBC parameters were derived based on the Beckman–Coulter method of counting and sizing in conjunction with an automated diluting and mixing apparatus for sample processing. The NLR was calculated for each participant by dividing the total absolute neutrophil count by the total absolute lymphocyte count. Details of the laboratory methodology are available at: https://wwwn.cdc.gov/Nchs/Nhanes/2013-2014/CBC_H.htm

#### 2.2.2 AAC

AAC was evaluated in this study using the Kauppila AAC score, which was obtained from dual-energy x-ray absorptiometry (DXA) scans of the lateral lumbar spine (12). The calcification severity was indicated by the AAC score, with a total AAC score ranging from 0 to 24. Higher AAC scores indicated more severe calcification and an AAC score of >6 is usually used as the cutoff for severe AAC (13). We considered the NLR as a continuous variable and used a three-category variable in our analysis to determine the relationship between the NLR and AAC.

### 2.3 Covariables

The covariates included sex, age (<65 or ≥65 years), race, income-to-poverty ratio (PIR) (<1.3 or ≥1.3), education level (high school and below or above high school), body mass index (BMI) (<25, 25–30, or ≥30 kg/m^2^), drinking, smoking, hypertension, diabetes mellitus (DM), coronary heart disease (CHD), angina pectoris, heart failure (HF), chronic kidney disease (CKD), stroke, triglyceride, total cholesterol, platelet count (PLT), blood urea nitrogen (BUN), serum creatinine (SCr), serum uric acid (SUA), serum total calcium (SCa), and serum phosphorus (SPh) levels.

### 2.4 Statistical analysis

All normally distributed continuous variables are reported as means ± SD, whereas skewed continuous variables are presented as medians [interquartile range (IQR)]. Categorical variables are expressed as frequencies and percentages. Statistical analyses included the use of the chi-square or Fisher's exact tests for categorical variables, one-way analysis of variance (ANOVA) for variables with a normal distribution, and the Kruskal–Wallis H test for variables with a skewed distribution to assess differences among the various NLR groups. Multiple imputations were used to fill in missing baseline data.

Multinomial logistic regression was conducted to investigate the association between the NLR and AAC. The NLR was considered both a continuous and a categorical variable, comprising the following categories: Q1 (<1.64), Q2 (1.64–2.34), and Q3 (>2.34). These categories were selected based on clinical interest and literature (14–16). Three models were constructed in this study. Model 1 was unadjusted. Model 2 was adjusted for sex, age, race, PIR, and education level. Model 3 was additionally adjusted for BMI, alcohol consumption, smoking, hypertension, DM, CHD, angina pectoris, HF, CKD, stroke, triglycerides, total cholesterol, PLT, BUN, SCr, SUA, SCa, and SPh levels.

A multivariate regression model was employed for trend analysis, with the NLR utilized as both continuous and three-category variables. A restricted cubic spline model was used to generate smooth curves and investigate the potential non-linear dose-response relationships between the NLR and SAAC. Subgroup analysis was conducted to investigate the association between the NLR and AAC based on the identified subgroup variables. All statistical analyses were conducted using R Statistical Software, version 4.2.2 (http://www.R-project.org, The R Foundation) and the Free Statistics Analysis Platform (17), version 2.0 (Beijing, China; http://www.clinicalscientists.cn/freestatistics). Statistical significance was defined as a two-sided *p* < 0.05.

## 3 Results

After rigorous screening based on the predefined inclusion and exclusion criteria, this study included a total of 3,047 patients (1,469 males and 1,578 females) (Figure 1). The overall prevalence of severe calcification disease in the study population was 10.8%. The participants were classified into three quantiles based on their NLR: quantile 1 (<1.64%), quantile 2 (1.64%–2.34%), and quantile 3 (>2.34%). The baseline patient characteristics are shown in Table 1. Sex, age, race, BMI, alcohol consumption, smoking, hypertension, DM, CHD, angina pectoris, HF, CKD, stroke, triglyceride levels, total cholesterol, BUN, SPh, and SCr differed significantly between the three NLR groups, while PIR, PLT, SUA, and SCa did not (all *p* > 0.05).

**Table 1:** Characteristics of the study population based on NLR tertiles.

Variables	Total (*n* = 3,047)	NLR			*P* value
		Q1 (*n* = 1,016)	Q2 (*n* = 1,015)	Q3 (*n* = 1,016)	
Sex, *n* (%)					0.007
Male	1,469 (48.2)	452 (44.5)	495 (48.8)	522 (51.4)	
Female	1,578 (51.8)	564 (55.5)	520 (51.2)	494 (48.6)	
Age, *n* (%)					<0.001
<65	2,045 (67.1)	732 (72)	710 (70)	603 (59.4)	
≥65	1,002 (32.9)	284 (28)	305 (30)	413 (40.6)	
Race, *n* (%)					<0.001
Mexican American	402 (13.2)	113 (11.1)	152 (15)	137 (13.5)	
Other Hispanic	289 (9.5)	103 (10.1)	106 (10.4)	80 (7.9)	
Non-Hispanic White	1,349 (44.3)	329 (32.4)	451 (44.4)	569 (56)	
Non-Hispanic Black	586 (19.2)	294 (28.9)	163 (16.1)	129 (12.7)	
Other Race	421 (13.8)	177 (17.4)	143 (14.1)	101 (9.9)	
Educational level, *n* (%)					0.158
High school and below	1,389 (45.6)	487 (47.9)	457 (45)	444 (43.8)	
Above high school	1,658 (54.4)	529 (52.1)	558 (55)	571 (56.2)	
PIR, *n* (%)					0.913
<1.3	934 (30.7)	314 (30.9)	306 (30.1)	314 (30.9)	
≥1.3	2,113 (69.3)	702 (69.1)	709 (69.9)	702 (69.1)	
BMI, *n* (%)					0.008
<25	974 (32.0)	357 (35.1)	296 (29.2)	321 (31.6)	
25–30	1,158 (38.0)	360 (35.4)	426 (42.0)	372 (36.6)	
>30	915 (30.0)	299 (29.4)	293 (28.9)	323 (31.8)	
Smoke, *n* (%)					<0.001
Yes	1,407 (46.2)	425 (41.8)	447 (44)	535 (52.7)	
No	1,640 (53.8)	591 (58.2)	568 (56)	481 (47.3)	
Alcohol consumption, *n* (%)					<0.001
Yes	2,170 (71.2)	674 (66.3)	753 (74.2)	743 (73.1)	
No	877 (28.8)	342 (33.7)	262 (25.8)	273 (26.9)	
Hypertension, *n* (%)					0.030
Yes	1,446 (47.5)	457 (45)	474 (46.7)	515 (50.7)	
No	1,601 (52.5)	559 (55)	541 (53.3)	501 (49.3)	
Diabetes, *n* (%)					0.005
Yes	506 (16.6)	140 (13.8)	163 (16.1)	203 (20)	
No	2,421 (79.5)	831 (81.8)	815 (80.3)	775 (76.3)	
Boundary	120 (3.9)	45 (4.4)	37 (3.6)	38 (3.7)	
CHD, *n* (%)					<0.001
Yes	159 (5.2)	38 (3.7)	36 (3.5)	85 (8.4)	
No	2,888 (94.8)	978 (96.3)	979 (96.5)	931 (91.6)	
Angina pectoris, *n* (%)					0.004
Yes	96 (3.2)	27 (2.7)	22 (2.2)	47 (4.6)	
No	2,951 (96.8)	989 (97.3)	993 (97.8)	969 (95.4)	
HF, *n* (%)					<0.001
Yes	104 (3.4)	25 (2.5)	24 (2.4)	55 (5.4)	
No	2,943 (96.6)	991 (97.5)	991 (97.6)	961 (94.6)	
CKD, *n* (%)					0.037
Yes	117 (3.8)	29 (2.9)	37 (3.6)	51 (5)	
No	2,930 (96.2)	987 (97.1)	978 (96.4)	965 (95)	
Stroke, *n* (%)					<0.001
Yes	131 (4.3)	28 (2.8)	37 (3.6)	66 (6.5)	
No	2,916 (95.7)	988 (97.2)	978 (96.4)	950 (93.5)	
Severe AAC, *n* (%)					<0.001
Yes	329 (10.8)	79 (7.8)	88 (8.7)	162 (15.9)	
No	2,718 (89.2)	937 (92.2)	927 (91.3)	854 (84.1)	
Triglyceride, mean ± SD	1.4 ± 1.0	1.4 ± 1.1	1.5 ± 1.0	1.4 ± 0.8	0.044
Total cholesterol, mean ± SD	5.0 ± 1.1	5.2 ± 1.2	5.1 ± 1.0	4.9 ± 1.1	<0.001
WBC, mean ± SD	7.1 ± 2.2	6.5 ± 1.8	7.0 ± 1.8	8.0 ± 2.5	<0.001
NLR, mean ± SD	2.2 ± 1.3	1.3 ± 0.3	2.0 ± 0.2	3.5 ± 1.7	<0.001
RBC, mean ± SD	4.6 ± 0.5	4.6 ± 0.5	4.7 ± 0.5	4.6 ± 0.5	0.001
PLT, mean ± SD	231.0 ± 59.5	229.6 ± 58.0	231.1 ± 57.4	232.1 ± 63.1	0.641
BUN, mean ± SD	5.1 ± 2.3	4.8 ± 1.9	5.0 ± 2.0	5.5 ± 2.7	<0.001
SCr, mean ± SD	83.4 ± 46.1	80.3 ± 31.4	81.6 ± 51.6	88.4 ± 51.9	<0.001
SUA, mean ± SD	324.2 ± 82.8	323.8 ± 83.4	320.5 ± 78.4	328.3 ± 86.2	0.107
SCa, mean ± SD	2.4 ± 0.1	2.4 ± 0.1	2.4 ± 0.1	2.4 ± 0.1	0.067
SPh, mean ± SD	1.2 ± 0.2	1.2 ± 0.2	1.2 ± 0.2	1.2 ± 0.2	<0.001

NLR, neutrophil-to-lymphocyte ratio; PIR, income-to-poverty ratio; BMI, body mass index; CHD, coronary heart disease; HF, heart failure; CKD, chronic kidney disease; AAC, abdominal aortic calcification; WBC, white blood cell; RBC, red blood cell; PLT, platelet count; BUN, blood urea nitrogen; SCr, serum creatinine; SUA, serum uric acid; SCa, serum total calcium; SPH, serum phosphorus.

### 3.1 Association between the NLR and SAAC

**Figure 2:**
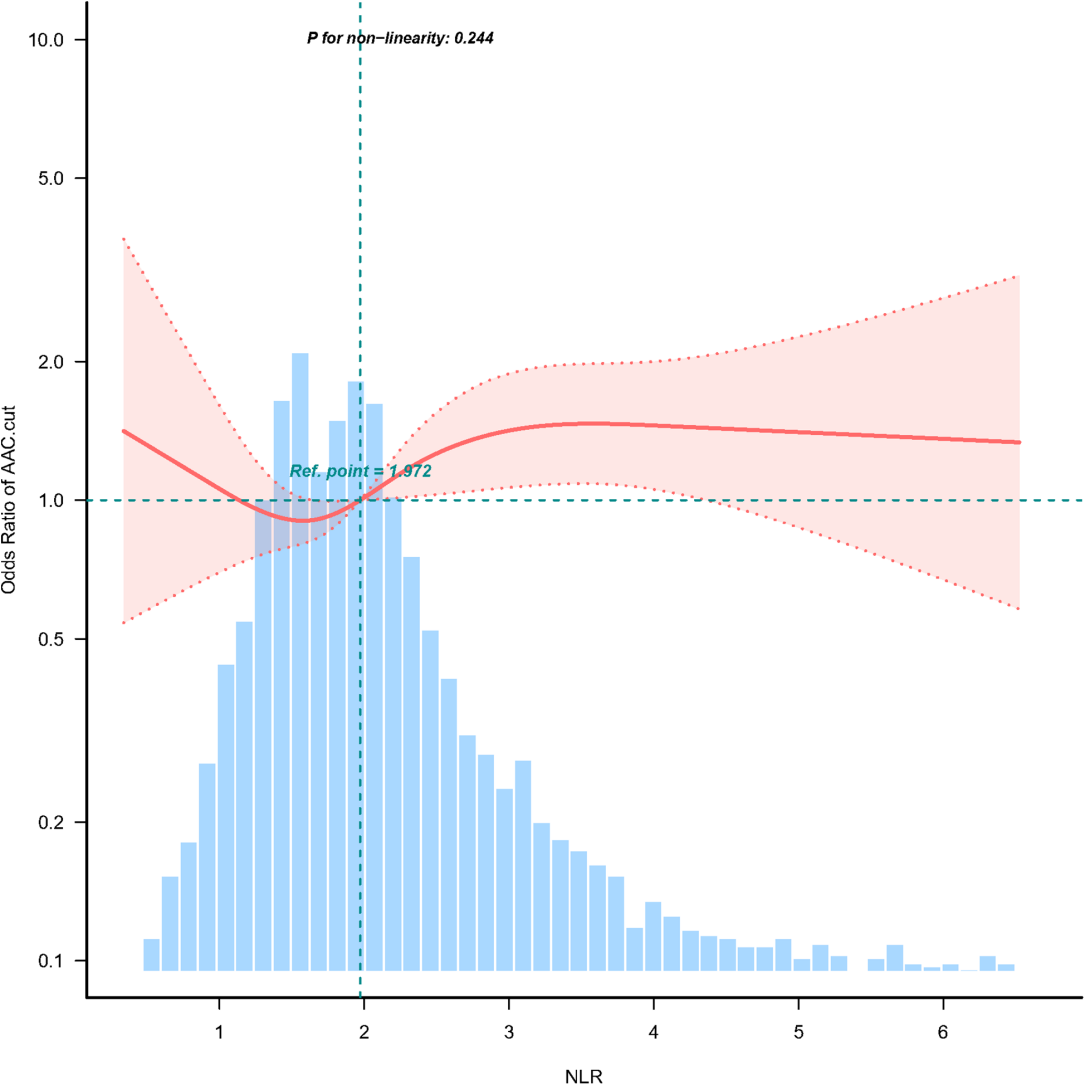
Association between NLR and SAAC odds ratio. Solid and dashed lines represent the predicted values and 95% confidence intervals. They were adjusted for sex, age, race, PIR, education level, BMI, alcohol consumption, Smoking, Hypertension, DM, CHD, Angina pectoris, HF, CKD, Stroke, triglycerides, total cholesterol, PLT, BUN, SCr, SUA, SCa, and SPh levels. Only 99% of the data is shown.

Multivariable logistic regression analyses adjusted for potential confounders (Table 2, Model 3), NLR expressed as a continuous variable was positively associated with the probability of SAAC [odds ratio (OR) = 1.08, 95% confidence interval (CI) = 1.00–1.16, *p* = 0.049]. The multivariable-adjusted regression ORs (95% CI) of NLR associated with groups 2 and 3 were 1.04 (95% CI = 0.73–1.48) and 1.42 (95% CI = 1.02–1.96), respectively, compared with NLR group 1 (Table 2, Model 3). The NLR was considered a continuous variable in the restricted cubic spline analysis. The results of multivariable-adjusted restricted cubic spline analysis revealed a linear relationship between the NLR and SAAC (Figure 2, *p* for non-linearity = 0.244, excluding the highest 1% for each NLR measure). The observed trend indicated that as the NLR increased, the risk of SAAC also increased.

**Table 2:** Multivariable logistic regression to assess the association of NLR with severe AAC.

Variable	Model 1		Model 2		Model 3	
	OR_95CI	*P* value	OR_95CI	*P* value	OR_95CI	*P* value
NLR (continuous variable)	1.26 (1.17∼1.35)	<0.001	1.15 (1.06∼1.24)	<0.001	1.08 (1.00∼1.16)	0.049
NLR (quartile)						
Q1 (<1.64)	1 (Ref)		1 (Ref)		1 (Ref)	
Q2 (1.64–2.34)	1.13 (0.82∼1.55)	0.463	1.04 (0.74∼1.45)	0.830	1.04 (0.73∼1.48)	0.825
Q3 (>2.34)	2.25 (1.69∼2.99)	<0.001	1.68 (1.23∼2.29)	0.001	1.42 (1.02∼1.96)	0.037

### 3.2 Subgroup analysis

**Figure 3:**
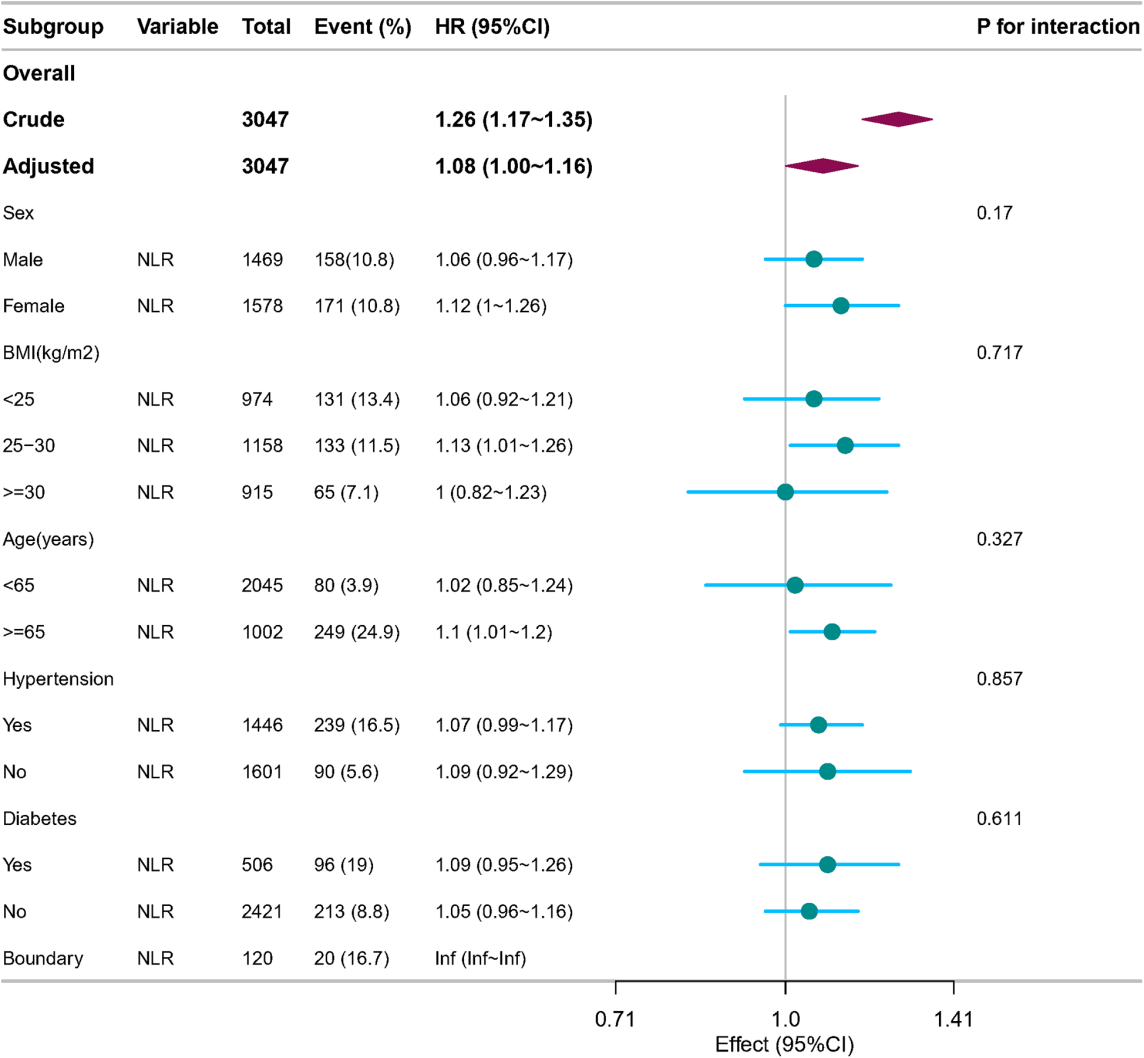
Forest plot of multivariable logistics analysis between NLR and SAAC. Except for the stratification component itself, each stratification factor was adjusted for sex, age, race, PIR, education level, BMI, alcohol consumption, Smoking, Hypertension, DM, CHD, Angina pectoris, HF, CKD, Stroke, triglycerides, total cholesterol, PLT, BUN, SCr, SUA, SCa, and SPh levels.

Subgroup analyses were conducted to examine the association between the NLR, incident AAC, and SAAC. These analyses were stratified by age, sex, hypertension, DM, and BMI. The results demonstrated consistent associations between NLR and SAAC across all subgroups and sensitivity analyses, with no statistically significant differences observed (all p-values for interaction >0.05) (Figure 3).

## 4 Discussion

The results of this comprehensive retrospective cross-sectional study, which analyzed data from the 2013–2014 NHANES, demonstrated that the NLR is an independent predictor of SAAC. This association was consistent across various clinical subgroups. Our findings have several important clinical implications.

Inflammation is strongly associated with the risk of cardiovascular mortality (18). The CANTOS trial (Canakinumab Anti-Inflammatory Thrombosis Outcomes Study) showed that targeted anti-inflammatory therapy—at least with interleukin-1ß inhibition—could significantly lower the rates of heart attack and cardiovascular death. Among patients at “residual inflammatory risk,” anti-inflammatory therapy reduces the risk of cardiovascular events (19, 20). Inflammatory markers, which are simple, measurable biological indicators, are important in guiding the long-term follow-up treatment of patients at high cardiovascular risk.

In the inflammatory expression stage, neutrophils suggest non-specific inflammation, while lymphocytes have defense and regulatory roles in the inflammatory response. NLR, as a combination of the two, can more comprehensively reflect the inflammatory state and immune status of the organism. This study investigated the non-linear relationship between the NLR and SAAC using data from the NHANES 2013–2014. Our findings are consistent with those of previous observational studies. For instance, Zhou et al. (14) reported a significant correlation between the NLR and elevated SAAC in a specific population (*β* = 2.37; 95% CI = 1.79–3.42, *p* = 0.025). Similarly, a recent prospective longitudinal cohort study involving patients with end-stage renal disease (ESRD) also demonstrated a significant association between the NLR and SAAC (21). Moreover, NLR is an independent predictor of thoracic peri-aortic calcification (TAC) in patients with ESRD. A recent study employing Mendelian randomization further confirmed these associations (22). These findings illustrate the potential of the NLR in risk stratification of patients with SAAC.

Further research is required to validate these results, investigate the detailed relationship between the NLR and SAAC, and elucidate the potential underlying mechanisms of this relationship. Recent studies have begun to unravel the relationship between the NLR and SAAC. For example, a cross-sectional analysis of baseline data from a multicenter cohort revealed the independent association between the highest tertile of NLR with AAC (OR = 2.876, 95% CI = 1.250–6.619, *p* = 0.013) (15). Our study extends these findings by demonstrating that a high NLR is associated with an increased risk of developing SAAC. Furthermore, our results indicated that with increased NLR, the risk of SAAC increased by 8% in the NHANES 2013–2014 dataset.

The correlations between the NLR and incident SAAC, although slight, were consistent across diverse event subtypes. These correlations persisted across the various subgroups defined by age, race, BMI, DM, and hypertension. These significant associations with SAAC are consistent with findings in the existing literature. Therefore, the results of the subgroup analyses demonstrate the overall stability of the findings.

A comprehensive understanding of the potential mechanisms and clinical implications is essential for elucidating the relationship between NLR and SAAC. Previous studies have suggested a metabolic link between the NLR and SAAC (23). Chronic inflammatory infiltration is often accompanied by osteochondral metaplasia and neovascularization (24). Moreover, the rate of hemodynamic progress is related to leukocyte chemotaxis and the expression of tumor necrosis factor-alpha (TNF-α) (25). Additionally, metalloproteinase (MMP) expression induces an inflammatory reaction (26). Finally, a variety of cytokines produced by inflammatory cells may accelerate and induce VC (27).

In summary, our study contributes to the growing body of evidence on the association between the NLR and SAAC. Further studies are required to gain a deeper understanding of the mechanisms and clinical implications of this relationship. This study represents an inaugural comprehensive investigation of the association between the NLR and the risk of developing AAC and cardiovascular events based on data obtained from the NHANES 2013–2014. The key strengths of this study are its large sample size, national population coverage, and representativeness, which provide robust statistical power to explore the relationship between the NLR and SAAC. The use of a territory-wide validated electronic healthcare database containing comprehensive records of diagnoses, hospitalizations, and medication dispensing details helped reduce the impact of common biases observed in observational studies, such as selection and recall biases (28).

The methodology in the present study was sound, demonstrating both novel and potential clinical applications. As a recently identified inflammatory marker, the NLR is easily calculated and readily available in clinical settings. An independent association was also identified between the increase in NLR and the incidence of severe AAC, thereby providing substantial evidence for identifying SAAC.

This study has some limitations. For instance, the cross-sectional nature of this study precludes the establishment of direct causality, and the possibility of residual confounding by unmeasured variables cannot be discounted. Despite adjusting for relevant confounders in the multivariate model, unknown residual confounders such as dietary factors or family income may have influenced the observed associations. Nevertheless, the homogeneity of the study population and adjustment for significant confounders suggests that any potential bias is likely minimal. Furthermore, due to the constraints of the NHANES database and the lack of data on AAC progression, a more comprehensive assessment of the relationship between NLR and AAC was not possible. Assessment of aortic calcification using DXA is less accurate than CT, but lateral spine images obtained with DXA to detect the prevalence of VFA can detect AAC with reasonably good sensitivity and specificity (29, 30). A high level of quality control was maintained throughout the DXA data collection and scan analysis (31) to ensure accuracy and consistency. In the future, further studies using more precise techniques are necessitated. Finally, only including the U.S. population aged >40 years limits the generalizability of our results. Further investigations across diverse populations are required to validate these findings.

In conclusion, the results of this study indicate a positive correlation between the NLR and SAAC in US adults aged >40 years. Thus, the NLR may be a novel inflammatory marker with implications for AAC.

## Data Availability

The raw data supporting the conclusions of this article will be made available by the authors, without undue reservation.
